# Innovative use of squid (*Loligo* spp.) ink powder as a potent immunostimulant for vannamei shrimp (*Litopenaeus vannamei*) in the treatment of infectious myonecrosis

**DOI:** 10.14202/vetworld.2025.1777-1788

**Published:** 2025-06-27

**Authors:** Mohamad Fadjar, Hartmut Kühn, Ayu Winna Ramadhani, Diana Aisyah, Cucun Herlina, Rangga Idris Affandi, Jefri Anjaini

**Affiliations:** 1 Department of Fisheries and Water Resource Management, Faculty of Fisheries and Marine Science, University of Brawijaya, Malang, Indonesia; 2 Department of Biochemistry, Charité-Universitätsmedizin Berlin, Berlin, Germany; 3 PSDKU Aquaculture, Faculty of Fisheries and Marine Science, University of Brawijaya, Malang, Indonesia; 4 Department of Fisheries and Marine Science, Faculty of Agriculture, University of Mataram, Mataram, Indonesia; 5 Department of Fisheries, Faculty of Fisheries and Marine Science, University of Jenderal Soedirman, Purwokerto, Indonesia

**Keywords:** aquaculture health, immunostimulant, infectious myonecrosis virus, *Litopenaeus vannamei*, non-specific immune response, squid ink powder

## Abstract

**Background and Aim:**

Infectious myonecrosis virus (IMNV) is a significant pathogen affecting *Litopenaeus vannamei*, causing high mortality and substantial economic losses in shrimp aquaculture. Conventional chemotherapeutics have limited efficacy and raise environmental concerns. This study explores the immunostimulatory potential of squid (*Loligo* spp.) ink powder as a natural dietary supplement to enhance the nonspecific immune responses in *L. vannamei* and mitigate IMNV-associated pathology.

**Materials and Methods:**

A completely randomized design was employed, with five groups: a negative control (healthy shrimp), a positive control (IMNV-infected), and three treatment groups that received squid ink powder at 400, 500, and 600 mg/kg feed, respectively. The feed was administered before and after IMNV immersion challenge. Immune parameters assessed included total hemocyte count (THC), differential hemocyte count (DHC), respiratory burst (RB), phenoloxidase (PO), superoxide dismutase (SOD), phagocytic activity, and ribonucleotide reductase (RR) expression. Statistical analysis was conducted using a one-way analysis of variance with Duncan’s *post hoc* test.

**Results:**

The 500 mg/kg dose of squid ink powder significantly enhanced shrimp immunity post-IMNV challenge. This treatment yielded the highest THC (6 × 10^5^ cells/mL), RB (1.13 optical density [OD]), SOD (0.98 units/mL), PO (0.619 OD), and phagocytic activity. A marked reduction in RR enzyme expression was observed, indicating effective viral suppression. DHC analysis revealed elevated granulocyte and semi-granulocyte counts, suggesting heightened immunological activity. Water quality parameters remained within acceptable aquaculture limits, and proximate analysis confirmed an improvement in protein content in the feed following supplementation.

**Conclusion:**

Squid ink powder at 500 mg/kg feed significantly enhances the non-specific immune system in *L. vannamei* and reduces IMNV-induced pathology. This natural additive offers a promising, sustainable alternative to synthetic immunostimulants in shrimp aquaculture.

## INTRODUCTION

Widespread disease outbreaks have consistently disrupted the shrimp aquaculture industry and remain one of its most significant threats. Infectious myonecro-sis virus (IMNV), a highly contagious viral disease in shrimp, has been reported in multiple countries and was first detected in *Litopenaeus vannamei* in Situbondo, East Java, in 2019. Due to the substantial economic losses associated with IMNV outbreaks, the development of effective disease control and prevention measures is critical for the sustainability of shrimp farming. In this industry, chemotherapeutic agents, particularly antimicrobials, are widely employ -ed to combat disease outbreaks [1]. Clinically, IMNV- infected vannamei shrimp exhibit signs of necrotic muscle tissues that discolor, beginning from a transparent hue to white and eventually turning reddish and decomposed, notably in the posterior segments near the tail base [2]. The disease initially presents acutely with high mortality in shrimp ponds and often progresses to a chronic phase, with mortality rates ranging between 40% and 70%. IMNV is believed to transmit vertically from broodstock to offspring and horizontally through waterborne exposure and cannibalistic behavior [3, 4].

To mitigate disease occurrence, various manage-ment strategies, including the use of immunostimulants, have been employed in aquaculture. In contrast to conventional approaches, the present study investigates squid ink powder as an innovative immunostimulant, providing a natural and eco-friendly alternative to synthetic compounds for enhancing the immune defenses of vannamei shrimp. Immunostimulants are agents that activate the host immune system, either partially or entirely, encompassing a wide range of substances. These include microbial cell wall derivatives such as lipopolysaccharides, β-glucans, and peptidoglycans, as well as probiotics, prebiotics, and other biologically derived extracts from plants or animals. These agents are typically used as prophylactics, with their most common mode of application being incorporation into formulated feeds, although they can also be directly introduced into aquaculture systems [5]. This study postulates that squid ink powder, rich in bioactive components, can enhance the immune system of *L. vannamei* and improve resistance to IMNV infection.

Squid (*Loligo* spp.) ink powder represents a promising alternative immunostimulant for enhancing host defenses against bacterial infections. Its nutritional profile is diverse, comprising 89.15% water, 9.37% protein, 0.85% carbohydrate, 0.28% fat, and 2.35% ash. Squid ink finds extensive applications in the pharmaceutical field due to its multifunctional bioactivity. It exhibits anti-inflammatory, antibacterial, antihypertensive, and antiretroviral properties and demonstrates antioxidant potential that may contribute to immune modulation [4]. While prior studies have predominantly concentrated on melanin as the primary active compound, ongoing research is exploring the broader chemical profile of squid ink. The pigment eumelanin found in squid ink comprises compounds such as 5,6-dihydroxyindole, 5,6-dihydroxyindole-2-carboxylic acid, and 2-carboxyl indole [6].

Although various immunostimulants have been explored to enhance the innate immunity of *L. vannamei* in aquaculture systems, there remains a paucity of natural, eco-friendly alternatives that provide broad-spectrum immunoprotection without contributing to antimicrobial resistance or environmental toxicity. Synthetic immunostimulants and chemotherapeutic agents, while effective, are often associated with high costs, limited availability, regulatory constraints, and potential residue accumulation in aquaculture environments. Moreover, the frequent reliance on antibiotics for disease prevention has raised global concerns regarding resistant pathogen strains and ecological imbalances [1, 5].

Squid (*Loligo* spp.) ink has gained attention in pharmacological research due to its diverse bioactive constituents, including melanin, alkaloids, peptides, and antioxidant compounds that exhibit antimicrobial, anti-inflammatory, and immunomodulatory properties [4, 6]. However, its application as a functional feed additive in shrimp aquaculture remains underexplored. To date, few studies have systematically evaluated the effect of squid ink powder on key non-specific immune parameters, such as hemocyte activity, respiratory burst (RB), superoxide dismutase (SOD), phenoloxidase (PO), and phagocytosis, in *L. vannamei*, particularly under viral challenge conditions, such as IMNV infection. In addition, the immunohistochemical analysis of ribonucleotide reductase (RR) expression as a marker for viral replication in treated shrimp has not been comprehensively investigated. Thus, there is a critical need to identify and validate sustainable, natural immunostimulants that can enhance host resilience to viral pathogens while maintaining environmental compatibility and production viability.

This study aims to investigate the immunostimulatory potential of squid (*Loligo* spp.) ink powder as a dietary supplement for *L. vannamei* challenged with IMNV. Specifically, the objectives are to (i) determine the optimal dosage of squid ink powder that elicits a significant enhancement in the shrimp’s non-specific immune response; (ii) assess the immunological markers including total and differential hemocyte counts (DHCs), RB, PO, SOD, and phagocytic activity; and (iii) evaluate viral suppression through RR expression using immunohistochemistry. By addressing these aims, the study seeks to provide a scientific basis for the integration of squid ink powder as a natural, sustainable immunostimulant in shrimp aquaculture.

## MATERIALS AND METHODS

### Ethical approval

The study was approved by University of Brawijaya Ethics Committee with Ethical Approval No. 026/EC/KEPK/09/2023.

### Study period and location

The study was conducted during August and November 2023 at the Integrated Research Laboratory and the Fish Disease and Health Disease, Faculty of Fisheries and Marine Science, University of Brawijaya.

### Experimental animals and design

**Figure 1:**
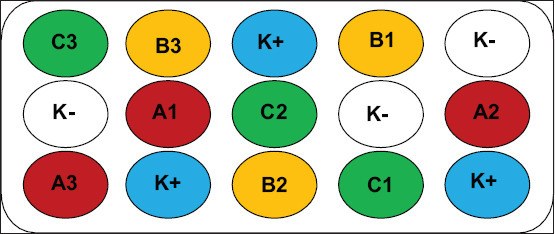
Research design. K(+) Positive control, K(-) Negative control, A: 400 mg squid ink powder/kg feed, B: 500 mg of squid ink powder per kg of feed, C: 600 mg of squid ink powder per kg of feed, 1, 2, 3: Repeated treatment.

The experimental fish were treated with different doses of squid ink powder (*Loligo* spp.) using 6–7 g vannamei shrimp at a density of 10 shrimp per 20 L aquarium. The squid were purchased from Fish Landing Place (Tempat Pelelangan Ikan) Lekok, Pasuruan, East Java. A completely randomized design was implemented, incorporating advanced immunological assays to assess the impact of squid ink powder on immune biomarkers in vannamei shrimp. The design consisted of three treatments and two controls, each with three replications: (1) Positive control (K+): IMNV-infected shrimp without squid ink powder, (2) negative control (K−): healthy shrimp without squid ink powder or IMNV infection, (3) treatment A: 400 mg squid ink powder/kg feed, (4) treatment B: 500 mg/kg feed, and (5) treatment C: 600 mg/kg feed. Squid ink powder was administered before and after IMNV infection (Figure 1). Dose selection was based on a lethal concentration 50 test, identifying 500 mg/kg as the concentration resulting in 50% shrimp mortality compared to the control. Measurements were taken at 4 time points: week 0 (baseline), week 1 (post-treatment), week 2 (IMNV onset), and week 3 (7 days post-infection).

### Experimental diet and feeding

The powder was prepared and applied to shrimp feed 4 times a day, comprising up to 5% of the daily biomass. A proximate test of mixed feed was conducted before and after the addition of squid ink powder using near-infrared reflectance spectroscopy. The Fourier transform near-infrared spectroscopy System monochromator (Bomem, Canada, Version 1, 1994) scanned samples in bounce mode across the range of 1,000–2,500 nm. A 20 g sample was placed in the measuring cell, and 64 consecutive scans were performed to determine the chemical composition.

### IMNV challenge test

Vannamei shrimp used in this study were obtained from a pond in Lamongan, East Java, and approved by the Animal Ethics Committee. After detecting the *RdRp* gene, the virus stock was prepared by mincing 1 g of IMNV-positive shrimp meat. This was diluted in 10 mL phosphate-buffered saline and centrifuged at 4,000 × *g* for 20 min at 4°C. The supernatant was further centrifuged at 18,000 × *g* (4°C) for 20 min and filtered with a 0.45 μm syringe filter. The final virus stock (1.02 × 10^7^ copies/mL) was stored at −70°C. IMNV infection was administered through immersion at a 1:100 dilution, based on the lethal dose 50 from preliminary tests. The challenge test lasted 14 days, as shrimp typically perish 1–15 days after the onset of clinical symptoms [1].

### Immune response parameters

#### Total hemocyte count (THC)

Using a 1 mL syringe, 50 μL of hemolymph was extracted from the ventral region of the shrimp’s second abdomen. An equal volume of 10% sodium citrate (pH 7.2) was added, followed by 100 μL of trypan blue. The sample was placed in a hemocytometer, and the number of cells/mL was counted using a 100× light microscope [7].

#### DHC

A hemolymph drop was placed on a glass slide, fixed with methanol for 5–10 min, and stained with Giemsa for 15–20 min. After rinsing and drying, preparations were examined using immersion oil and a 1,000× microscope (CX41, Olympus, CX 41RF, Japan). Granulocytes, semi-granulocytes, and hyaline cells were counted from 100 hemocytes [8].

#### RB

To perform RB measurement, 50 μL hemolymph was mixed with 10% Na-citrate and incubated at room temperature for 30 min. After centrifugation at 1,000 × *g* for 20 min, the supernatant was discarded. The precipitate was treated with 100 μL Nitro blue tetrazolium (NBT) in 0.3% Hank’s Balanced Salt Solution (HBSS) and left for 2 h. Following another centrifugation (10 min, 1,000 × *g*), the supernatant was discarded. The pellet was washed twice with 70% methanol after the addition of methanol and centrifugation. The precipitate was then dissolved in 120 μL potassium hydroxide (2M) and 140 μL dimethyl sulfoxide. Absorbance was measured at 630 nm using a microplate reader. RB values were expressed based on NBT reduction per 10 μL hemolymph [6].

#### SOD

For SOD analysis, 40 μL of hemolymph was combined with 10% Na-citrate and 360 μL of phosphate buffer (50 mM, pH 7.4), then centrifuged at 6,000 × *g* for 7 min at 4°C. The supernatant was heated at 65°C for 5 min, then reacted with 50 μL of NBT in 0.3% HBSS. Absorbance was read at 630 nm using a microplate reader (BiotTek 800TS, Agilent, USA) [5].

#### PO

PO activity was measured spectrophotometrically by recording dopachrome formation from L-dihydroxyphenylalanine (L-DOPA) [9].

#### Phagocytic activity

Phagocytic activity was measured by adding 25 μL of *Staphylococcus aureus* (10^7^ cells/mL) to 0.1 mL of hemolymph. After 20 min incubation, 5 μL was smeared onto a glass slide. Following fixation with methanol and Giemsa staining (10%, 15 min), the percentage of hemocytes undergoing phagocytosis was calculated [10].

#### RR

Histological samples underwent fixation, dehydration, clearing, embedding, sectioning, and mounting. Immunohistochemical staining was performed with anti-Rabbit Recombinant Monoclonal 2 primary antibody and fluorescein isothiocyanate-conjugated secondary antibody. Observations under a confocal microscope used a 488 nm wavelength, 30 μm scale, and 400× magnification. Green fluorescence indicated RR enzyme expression [11].

##### Water quality monitoring

Water quality parameters (dissolved oxygen, temperature, pH, salinity) were measured twice daily between 8 a.m. and 6 p.m. Weekly measurements of nitrate, nitrite, ammonia, total organic matter (TOM), and alkalinity were taken at weeks 0, 1, 2, and 3. Nitrate, nitrite, and ammonia were measured using a test kit (Prodactest™ NO_2_, NO_3_, NH_3_/NH_4_, PRO.D.AC International, Italy). TOM and alkalinity were assessed through titration.

#### Statistical analysis

All data were tested for normality before statistical analysis. A one-way analysis of variance was conducted using Statistical Package for the Social Sciences version 26 (IBM Corp., NY, USA) to determine significant differences among treatments. Where applicable, Duncan’s multiple range test was used to identify treatments that significantly improved the non-specific immune response in *L. vannamei*.

## RESULTS

### Proximate analysis of supplemented feed

The analysis of shrimp feed showed a notable increase in protein content after the addition of squid ink powder, rising from 37.12% in the control to a maximum of 40.62% (Table 1).

**Table 1:** Shrimp feed proximate analysis.

Treatment	Protein (%)	Fat (%)	Water (%)	Ash (%)	Raw fiber (%)
Shrimp feed (Control)	37.12	4.76	10.90	10.92	2.72
A (400 mg/kg)	40.38	4.03	10.43	12.50	2.90
B (500 mg/kg)	40.49	4.39	10.73	12.44	2.39
C (600 mg/kg)	40.62	4.18	10.90	12.40	2.52
Squid ink powder	10.25	1.35	0.35	1.28	1.11

### Immune response evaluation

#### THC

**Figure 2:**
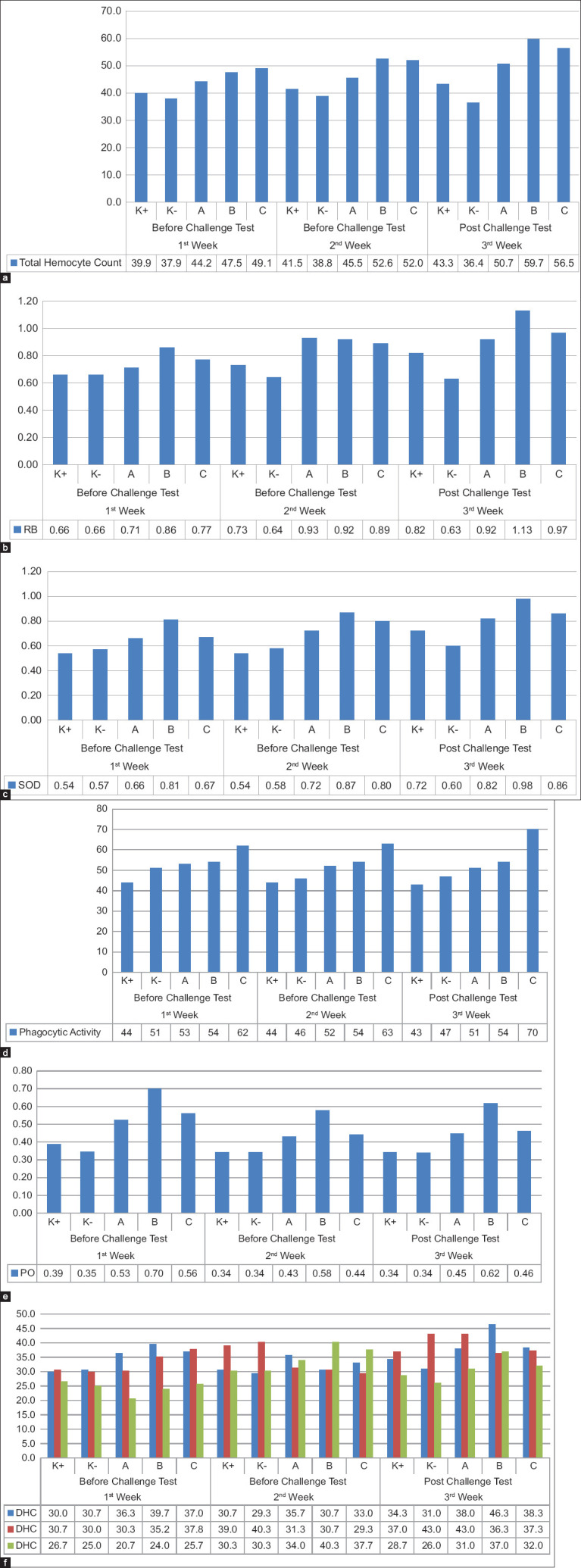
Effect of squid ink powder on immune response. (a) total hemocyte count, (b) respiratory burst, (c) superoxide dismutase, (d) phagocytic activity, (e) phenoloxidase, and (f) differential hemocyte count.

Treatment B (500 mg squid ink powder/kg feed) demonstrated a marked enhancement in immune response, exhibiting the highest THC value of 6 × 10^5^ cells/mL at 523.8 mg/kg feed. This result significantly outperformed treatments A (400 mg/kg) and C (600 mg/kg) as well as both control groups (Figure 2a).

#### RB

Treatment B significantly increased RB activity from baseline (week 0) to 7 days after IMNV infection (week 3). The positive control also showed an increase, reflecting the shrimp’s innate immune reaction to IMNV. A positive correlation was observed between squid ink dosage and RB activity, with a peak at 505.4 mg/kg resulting in an RB value of 1.1. Although treatment C showed similar results, treatment B reached the highest RB value of 1.13 optical density (OD), compared to 0.92 OD in treatment A and 0.97 OD in treatment C (Figure 2b), confirming the optimal immunostimulatory effect of 500 mg/kg.

#### SOD

Fourteen days after squid ink powder administration (week 2), the highest SOD value was recorded in treatment B (0.87 units/mL), which was significantly higher than in the other treatments and controls (Figure 2c). Post-infection measurements revealed sustained elevation in SOD levels in treatment B, peaking at 0.98 units/mL.

#### Phagocytic activity

Treatment B induced the most significant increase in phagocytic activity, demonstrating superior immune function relative to other treatments (Figure 2d). Elevated phagocytic activity reflects an effective immune response, as phagocytes engulf and neutralize pathogens.

#### PO activity

PO activity increased on the 1^st^ day following squid ink administration and IMNV infection. A decrease was noted on day 4 across all treatments. On day 4, treatment A had a PO value of 0.431 ± 0.005, and treatment C had a value of 0.442 ± 0.005. Treatment B recorded a significantly higher PO value of 0.578 ± 0.003 (p < 0.05). After 7 days, PO activity remained highest in treatment B (6.6%), followed by treatment C (4.12%) and control (4%) (Figure 2e), reinforcing the potent immunostimulant effect of the 500 mg/kg dosage.

#### DHC

Feeding shrimp with squid ink-supplemented diets resulted in a reduction in hyaline cells and an increase in granular cells (GCs), indicating the maturation of hemocyte subtypes. Treatment B showed the highest hyaline cell count post-infection at 46.3%, suggesting enhanced phagocytic activity. Semi-GCs (SGCs) increased notably in treatment C post-infection, while treatment B consistently outperformed other groups in GC counts, with 40.3% in week 2 and 37.0% in week 3 (Figure 2f). These results indicate a strong role of treatment B in promoting degranulation, cytotoxicity, and immune competence.

#### RR expression

**Figure 3:**
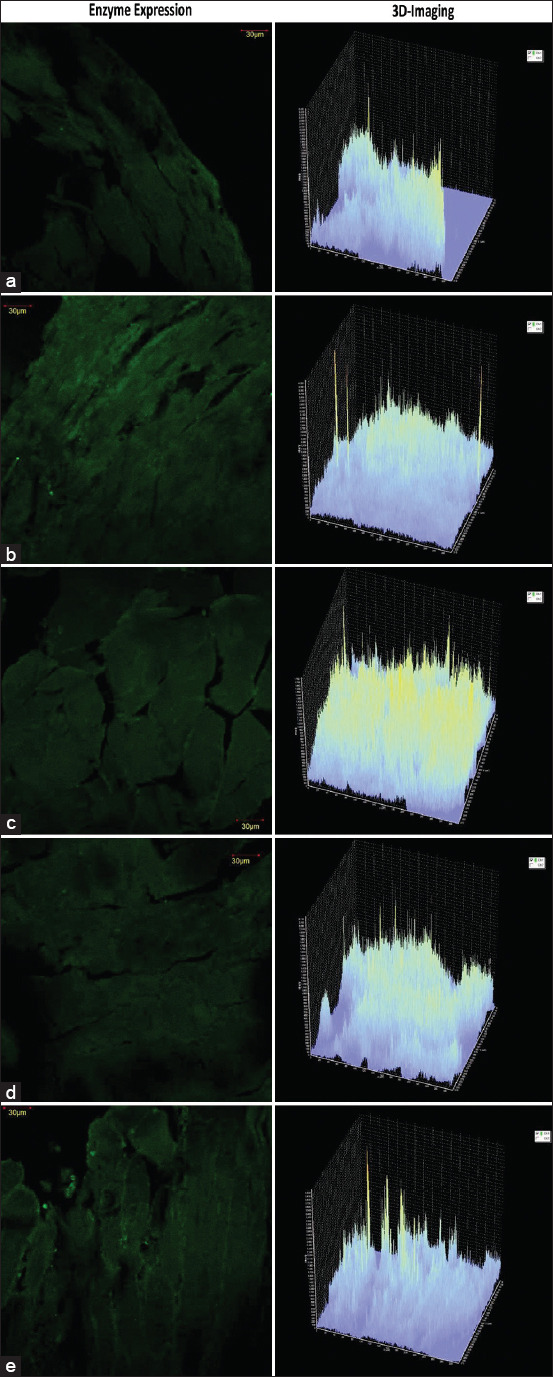
Ribonucleotide reductase enzyme expressed in vannamei shrimp (Litopenaeus vannamei). (a) K(+) (Positive control), (b) K(-) (Negative control), (c) treatment A (400 mg of squid ink powder/kg feed), (d) treatment B (500 mg of squid ink powder/kg feed), and (e) treatment C (600 mg of squid ink powder/kg feed).

Fluorescent imaging revealed that green luminescence intensity correlated with RR enzyme expression in shrimp muscle tissue (Figure 3). The highest RR expression was observed in the K+ group, attributable to the absence of squid ink supplementation, resulting in reduced immune resistance. Conversely, treated groups displayed reduced luminescence, indicating decreased RR activity. This reduction is linked to enhanced antibody production and hemocyte activation by the active compounds in squid ink, thereby increasing shrimp resistance to pathogens [7].

##### Water quality assessment

All monitored parameters remained within the optimal range for shrimp aquaculture, indicating that dietary supplementation with squid ink powder did not negatively affect water quality (Table 2) [6, 8, 10].

**Table 2:** Water quality parameters.

No.	Parameter	Measurement	References
1.	pH	6.82–8.9	7.5–8.5 [6]
2.	Salinity	16–18 ppt.	5–45 ppt [6]
3.	Temperature	25°C–34.3°C	25°C–32°C [8]
4.	Dissolved oxygen	4.2–9.4 ppm	> 4 ppm [8]
5.	Ammonia	0–1 ppm	Maximal value of 0.01 ppm [10]
6.	Nitrite	0.3–1.6 ppm	1.1–1.2 ppm [10]
7.	Total organic matter	0–32.79 ppm	Maximal 55 ppm [10]
8.	Alkalinity	24–508 ppm	100–150 [10]

## DISCUSSION

### Nutritional profile and dosage application

Shrimp can be protected from virus infections by adding squid ink as a diet supplement or extract. In this study, squid ink powder was used at concentrations of 400, 500, and 600 mg/kg, yielding crude protein contents of 40.38%, 40.49%, and 40.62%, respectively. Meanwhile, the cuttlefish ink extract at 500 mg, using hexane as the solvent, exhibited a moisture content of 4.43% ± 0.29%. Squid ink powder had a protein value of 62.46% ± 0.62%. The fat content of the squid ink powder was 3.96% ± 0.08%. A sample’s entire mineral content can be ascertained using ash analysis; 9.29% ± 0.05% of the total mineral content was found in squid ink powder [12]. Factors influencing the differences in protein levels include the solvent, concentration, and extraction process.

### Immune-stimulating properties of squid ink powder

This study is the first to demonstrate the immunostimulatory efficacy of squid ink powder in vannamei shrimp, revealing its multifaceted bioactive properties, including anti-inflammatory, antibacterial, antihypertensive, antiretroviral, and antioxidant activities, which collectively contribute to enhanced disease resilience. The immune-enhancing effects of squid ink powder were evident across multiple parameters, with the 500 mg/kg feed dose exhibiting the most significant improvement in non-specific immunity, as demonstrated by increased THC, DHC, RB, SOD, PO, and phagocytic activity, as well as reduced RR expression.

### Composition and antiviral activity of bioactive compounds

Qualitative analysis of squid ink revealed the abundant presence of proteins, phenols, amino acids, flavonoids, alkaloids, saponins, and carbohydrates in the methanolic extract. Conversely, steroids, terpenoids, tannins, and glycosides were found in smaller amounts, whereas anthraquinones, fixed oils, and fats were notably absent [13]. Squid ink contains alkaloids that can boost the nonspecific immune response and inhibit or stop the transcription of viruses, thereby reducing their reproduction [14]. Alkaloid substances such as IMNV can prevent the reproduction of RNA viruses. Alkaloid chemicals found in squid ink can raise THC. Alkaloids have antibacterial, anti-stress, immunostimulatory, and growth-promoting properties [15]. Alkaloids effectively destroy bacteria and viruses because they are harmful to microorganisms. Alkaloids suppress bacterial growth through multiple mechanisms, including interference with bacterial nucleic acid and protein synthesis, alteration of bacterial cell membrane permeability, damage to the cell membrane and cell wall, disruption of bacterial metabolic processes, and inhibition of efflux pumps [16]. These bioactive compounds act synergistically to reinforce the nonspecific immune defenses of shrimp and enhance resistance against viral infections.

### Hemocyte response and immune activation

Chemicals, medications, and other substances that can enhance both specific and non-specific response mechanisms are known as immunostimulants, such as those found in squid ink. The shrimp’s initial line of defense against infections is its non-specific immune system. The humoral immune system, phagocytosis, agglutination, and the synthesis of antimicrobial peptides are all reactions that the shrimp immune system produces in response to pathogen attacks [12]. Shrimp immune defenses rely on hemocytes, which play vital roles in both cellular and humoral immunity. In addition, the hemocyte count is a reliable indicator of the health status of penaeid shrimp. The ability of a chemical to activate the shrimp’s defensive mechanism was measured by the increase in hemocytes. Shrimp hemocytes are recognized as the primary line of defense against pathogenic microorganisms. The THC is easily influenced by both external (exogenous) and internal (endogenous) factors [17]. The results of this study showed that the squid ink THC values before and after infection in shrimp increased in response to IMNV. Hemocytes are frequently employed as quantitative indicators of shrimp stress. Shrimp immunity is determined by several factors, including THC. Normal white vannamei shrimp have between 20 × 10^6^ and 40 × 10^6^ cells/mL of THC [18].

### Recognition of immunostimulants and prophenoloxidase (proPO) activation

Immunostimulants are detected as foreign material when they enter the shrimp hemocoel. The pattern recognition proteins (PRPs) of shrimp hemocytes identify pathogen-associated molecular patterns (PAMPs) on immunostimulants. When a certain pattern is recognized, a chain of events occurs that includes phagocytosis, melanization, and the creation of a hostile environment for the pathogen. These investigations demonstrate that shrimp defense systems remain robust even when their immunological parameters return to their pre-stimulant levels, which facilitates the clearance of pathogens [19]. Foreign particles known as IMNV enter the body during infection, causing the semi-granular and granular hemocytes to release the activated proPO system. An increase in hemocytes may also elevate the number of GCs, which stimulate the activation of the ProPO system, leading to PO activity that protects shrimp from pathogenic attacks. Oral administration of acid and sodium alginate enhances ProPO system activity, thereby stimulating hemocyte production and phagocytosis [20]. This enzyme, which is involved in melanization, is produced by the proPO system and can be triggered by immunostimulants. The results of this study demonstrate a notable increase in the PO value after infection. However, the observed increase was not statistically significant, which may be attributed to several factors, including the concentration of the immunostimulant, duration of the maintenance period, and methodology of immunostimulant administration. Pathogens that infiltrate the shrimp’s body will be eliminated by melanin [21].

### Pattern recognition and melanization cascade

The shrimp innate system is activated by the presence of invading pathogens because it can distinguish between the “non-self” cells of an invading pathogen and “self-cells” belonging to itself. Shrimp hemocytes recognize and eliminate non-self-matter by recognizing specific patterns, known as PAMPs, located on pathogens. The receptors located on hemocytes that recognize these PAMPs are called host PRPs. Similar to any infection, immunostimulants are also detected in the hemocoel of shrimps by recognition of diversified PAMPs on the immunostimulants. After a successful recognition between PAMPs and PRPs, the serine proteinase cascade is triggered, and the PAMPs induce SGCs and GCs to degranulate and release granules containing the proPO system; then, proPO is converted to PO by active ppAE, resulting in the activation of melanization, release of cytotoxic compounds, and encapsulation [22].

### GCs and immune response indicators

Granule cells are characterized by a large number of granules, proPO, and cytotoxic activities. The function of GCs is more closely related to the production of phenol oxidase enzymes, which play a crucial role in non-specific defense systems. Prophenoloxidase is responsible for the production and secretion of toxic metabolites, such as quinones. All DHC parameters tested based on the criteria were found to be significantly increased. This is due to antigens entering the body. Then, the antigens are captured by macrophages in a manner that allows them to be recognized as foreign material. The foreign material is sent to the antibody-forming system, where antibody formation occurs [23]. The DHC results in the study using cuttlefish ink after infection were higher than the levels observed before infection.

### Role of reactive oxygen species (ROS), SOD, and RB in immunity

When shrimp become infected or suffer a stressor, hemocytes produce ROS, and their concentration is balanced with antioxidant enzymes, one of which is SOD. This increase in SOD reduces the explosion of cellular superoxide during the defense against viral infections and protects shrimp cells from damage. The RB is the basic form of the antibacterial system in the body of a fish. The increase in RB levels can be correlated with an increase in phagocytic cell activity [11]. The RB can increase oxygen consumption, resulting in the formation of superoxide anions, and this process is accelerated by NADPH oxidase, a multi-component enzyme that is attached to the inner surface of the plasma membrane after activation to carry out phagocytosis. The squid ink concentration will be balanced with antioxidant enzymes, one of which is SOD [7]. This increase in SOD reduces the explosion of cellular superoxide during the defense against viral infections and protects shrimp cells from damage [24]. Comparable results were observed in the squid ink study, where the SOD values increased after IMNV infection.

### RR and viral replication

The absence of IMNV infection in treated shrimp caused low RR expression in K-treatment. Viral replication is believed to be facilitated by the presence of the RR enzyme in living cells; the greater the RR expression intensity, the greater the viral replication in cells [25]. As observed by the rising intensity of RR expression in shrimp, the shrimp’s decreased immunity makes them resistant to infectious illnesses, which is linked to increased viral replication. It takes a long time for the shrimp’s immunological response if the active ingredient in the form of squid ink powder is administered at a low dose [26]. The ability of squid ink to modulate key immune markers and inhibit viral progression highlights its potential as a functional feed additive in shrimp aquaculture.

### Antimicrobial functions of squid ink bioactives

Squid uses its defense mechanism (squid ink) to fend off predator attacks by rupturing peptidoglycan components and blocking the enzyme topoisomerase, which is crucial for DNA replication, transcription, and recombination. These alkaloids, composed of nitrogen and its derivatives such as phenol, amine, amide, and methoxy, serve as potent antibacterial and antiviral agents [27]. An alkaloid found in squid (*Loligo* spp.) ink powder helps boost non-specific immune responses and decrease viral multiplication by preventing or reducing viral transcription. Choline (an alkaloid), cinnamic acid (a carboxylic acid), and betaine (an alkaloid) are the main constituents of squid ink powder. Their biological activities include antifungal, antiviral, antioxidant, and antibacterial activities. This result demonstrates that squid ink powder can be utilized to boost shrimp’s immune systems in aquaculture operations [28]. It takes less of an immunostimulant at low doses to elicit an immunological response in shrimp. Compared with shrimp infected with IMNV that did not receive immunostimulant treatment, those that received immunostimulants as protection against IMNV attacks had a higher survival rate. According to the previous study by Idris Affandi *et al*. [29], which employed various chemicals, squid ink powder can be used as an ingredient against IMNV, with outcomes that are no less effective than those of other compounds. Therefore, alkaloids and other active compounds in squid ink play crucial roles as antibacterial and antiviral agents for fish and shrimp.

### Comparative efficacy and environmental sustainability

In shrimp (*L. vannamei*), squid ink powder enhances non-specific immune responses by incr-easing hemocyte counts, modulating immune-related enzymes, and potentially suppressing viral multiplication [30, 31]. These effects may result from interactions with membrane receptors or intracellular pathways, although evidence of a unique biochemical mechanism remains inconclusive. Compared with synthetic immunostimulants such as Tian-Dong-Tang-Gan Powder and Fitoimun (Bioperkasa, Indonesia), which also boost PO, acid phosphatase, and SOD activity [31, 32], squid ink offers additional benefits through its complex and multifunctional bioactive profile. Environmentally, squid ink powder presents a more sustainable alternative; its naturally derived compounds are biodegradable and less likely to persist in aquaculture systems, unlike synthetic agents that may accumulate and disrupt aquatic ecosystems. Furthermore, natural immunostimulants may improve water quality by reducing residual waste [33]. Thus, squid ink powder is a promising eco-friendly immunostimulant, though further research is needed to elucidate its molecular mechanisms and long-term environmental impacts.

### Limitations and future directions

Although this study provides compelling evidence of the immunostimulatory potential of squid ink powder, some limitations should be acknowledged. First, the long-term effects of squid ink supplementation remain unexplored, necessitating further research on its prolonged administration and potential cumulative effects. Second, although the study confirmed a reduction in RR expression, additional molecular studies could further elucidate the exact antiviral mechanisms involved [12]. Future research should also explore the potential synergistic effects of squid ink powder with other immunostimulants and assess its impact under commercial shrimp farming conditions [18]. Future studies should focus on optimizing the formulation, dosage, and application methods to maximize the effectiveness of this approach in large-scale aquaculture operations.

## CONCLUSION

This study provides compelling evidence that dietary supplementation with squid (*Loligo* spp.) ink powder significantly enhances the non-specific immune response of *L. vannamei* against IMNV. Among the tested concentrations, the 500 mg/kg feed dosage proved to be the most effective, as evidenced by marked increases in THC, DHC, RB, SOD, PO, and phagocytic activity. Moreover, a concomitant reduction in RR expression indicated the suppression of viral replication at the cellular level. The proximate analysis of shrimp feed revealed enhanced protein content following squid ink supplementation, without compromising water quality, a critical parameter in sustainable aquaculture.

The major strength of this study lies in its compr-ehensive immunological assessment across cellular, enzymatic, and molecular biomarkers, combined with rigorous experimental controls and replication. In addition, the identification of squid ink’s multifunctional bioactive compounds – such as alkaloids and phenolics – provides a biochemical rationale for its immunostimulatory effects. Importantly, this study is among the first to correlate reduced RR expression with improved antiviral defense in shrimp following natural feed-based intervention.

Practically, the use of squid ink powder presents a cost-effective, biodegradable, and environmentally sustainable alternative to synthetic immunostimulants and antibiotics. Its application in commercial shrimp farming may reduce reliance on chemotherapeutics, mitigate risks of antimicrobial resistance, and improve survival and productivity during viral outbreaks.

In conclusion, squid ink powder at a dose of 500 mg/kg is a promising candidate for functional feed development in shrimp aquaculture. Future research should aim to elucidate the long-term safety, synergistic effects with other bioactives, and molecular pathways involved in its antiviral action. Its integration into large-scale farming systems holds significant promise for advancing sustainable and disease-resilient shrimp production.

## DATA AVAILABILITY

Datasets generated in this study are available from the corresponding author upon request.

## AUTHORS’ CONTRIBUTIONS

MF: Conceptualization, design, and management of the study. HK: Conceptualized the study. AWR, DA, and CH : Statistical analysis and drafted and revised the manuscript. RIA and JA: Conducted the study and collected the samples and data. All authors have read and approved the manuscript.
